# A case report of pneumomediastinum in a COVID‐19 patient treated with high‐flow nasal cannula and review of the literature: Is this a “spontaneous” complication?

**DOI:** 10.1002/ccr3.4007

**Published:** 2021-04-09

**Authors:** Anna Cancelliere, Giada Procopio, Maria Mazzitelli, Elena Lio, Maria Petullà, Francesca Serapide, Maria Chiara Pelle, Chiara Davoli, Enrico Maria Trecarichi, Carlo Torti, The IDTM UMG COVID‐19 Group

**Affiliations:** ^1^ Infectious and Tropical Disease Unit Department of Medical and Surgical Sciences “Magna Graecia” University of Catanzaro Catanzaro Italy; ^2^ Radiology Unit Department of Medical and Surgical Sciences “Magna Graecia” University of Catanzaro Catanzaro Italy

**Keywords:** acute hypoxemic respiratory failure, COVID‐19, high‐flow nasal cannula, oxygen support, SARS‐CoV‐2, spontaneous pneumomediastinum

## Abstract

Oxygen support with high‐flow nasal cannula (HFNC) is gentler than mechanical ventilation and may provide significant benefits, but more studies are needed to investigate the efficacy and safety of different respiratory supports in patients with COVID‐19 pneumonia.

## Graphical abstract

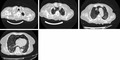

## 1 INTRODUCTION

The novel coronavirus (SARS‐CoV‐2) infection can lead to refractory hypoxemic respiratory failure and acute respiratory distress syndrome with possible fatal outcome. Patients can significantly benefit from high‐flow nasal cannula (HFNC) oxygen therapy. We report herein a case of pneumomediastinum, which could be a rare complication of HFNC, as well as of SARS‐CoV‐2 pneumonia. The patient, an 89‐year‐old woman without known conditions predisposing to pneumomediastinum, was admitted for SARS‐CoV‐2 pneumonia. During the following days, the patient conditions worsened notwithstanding the respiratory support and treatment applied; therefore, we decided to start oxygen therapy with HFNC. Despite respiratory improvement, the patient began to complain of chest pain, which increased with respiratory acts and radiated to the neck, especially along the left side. After diagnosis of pneumomediastinum, was made HFNC was suspended and a low‐flow system with nasal cannulas was started. Patient conditions progressively improved with clinical and radiological resolution of pneumomediastinum; subsequently, the patient completely recovered and was discharged COVID‐19 free. To our best knowledge, this is the second case of pneumomediastinum in a COVID‐19 patient after HFNC. From a review of similar cases and pathogenetic hypothesis, we can conclude that pneumomediastinum was caused by a combination of many factors (such as viral infection, cytokine storm, increase in transalveolar pressure due to cough, and possibly HFNC). Since pneumomediastinum is a severe complication, it is important to perform a prompt diagnosis, while treatment guidelines should define what is the best approach for oxygen support in COVID‐19 patients.

SARS‐CoV‐2 is a new coronavirus responsible for coronavirus disease 2019 (COVID‐19), which originated in the city of Wuhan, Hubei Province, Central China, and spreading quickly as a pandemic.^1^ This disease has many clinical presentations; indeed, it may be asymptomatic or it may present with typical symptoms such as fever, shortness of breath, cough, and loss of taste or with atypical presentations such as altered mental status, diarrhea, and nausea. In severe diseases, septic shock, acute respiratory distress syndrome (ARDS), difficult‐to‐correct metabolic acidosis, and coagulation dysfunction develop rapidly.^2^

The virus replicates rapidly upon entry into alveolar epithelial cells, and triggers a strong immune response, resulting in a cytokine storm syndrome and pulmonary tissue damage. Cytokine storm syndrome is a group of disorders characterized by the uncontrolled production of pro‐inflammatory cytokines, causing ARDS.^3^ Increased cytokines are released, such as interleukin (IL)‐6, and various acute‐phase reactants can lead to endothelial injury. Also, immobilization and hypercoagulable state due to elevated circulating prothrombotic factors, which are all elements/items included in Virchow's triad, can explain the high risk of coagulopathy.^4^

Multiple patchy ground‐glass opacities with peripheral distribution are typical chest features of the COVID‐19 pneumonia at computerized tomography (CT).^5^ These findings are mainly seen in the early stages of COVID‐19 pneumonia and may be attributed to alveolar swelling, exudation in the alveolar space, and alveolar septal inflammation.^6^

Spontaneous pneumomediastinum is an uncommon but usually benign and self‐limiting condition which most often occurs with chest pain, sometimes combined with dyspnea. This complication is generally caused by sudden increase in the thoracic pressure where the air dissects along the bronchovascular structures into the mediastinum resulting in alveolar rupture. Pulmonary diseases and smoking may also predispose to this condition.^7^ The precipitating factors may be forceful coughing or sneezing, but also several pulmonary infections (reviewed below in the paper). It is conceivable that SARS‐CoV‐2, which infects both type I and II pneumocytes, may cause breakdown of the integrity of the alveolar membrane, thus leading to alveolar rupture both via direct and indirect mechanisms mediated by hyperinflammation.^8^, ^9^

Severe COVID‐19 often progresses to acute hypoxemic respiratory failure requiring high fractional concentration of inspired oxygen (FiO2). High‐flow nasal cannula (HFNC) has emerged as a strategy improving oxygenation and carbon dioxide clearance. Through this procedure, the patient inspiratory demands were fulfilled by delivering up to 60 L/min of gas flow with an FiO2 up to 1.0, thus decreasing adverse outcomes, including the risk of dispersion of aerosolized particles relative to other techniques such as invasive ventilation.^10^ However, since COVID‐19 is a new entity, it is important to study efficacy and possible adverse events of HFNC more in depth.

We report herein a case of pneumomediastinum which occurred in a patient suffering from SARS‐CoV‐2 pneumonia and acute hypoxemic respiratory failure treated with HFNC. We also provided a review of other reported cases of pneumomediastinum in patients with COVID‐19.

## 2 CASE PRESENTATION

The patient was an 89‐year‐old woman, who never smoked. She was admitted to our ward complaining of dry cough, which later became productive accompanied by shortness of breath. A nasal pharyngeal swab for PCR detecting SARS‐CoV‐2 RNA resulted positive. No underlying pulmonary diseases were known before COVID‐19.

**FIGURE 1:**
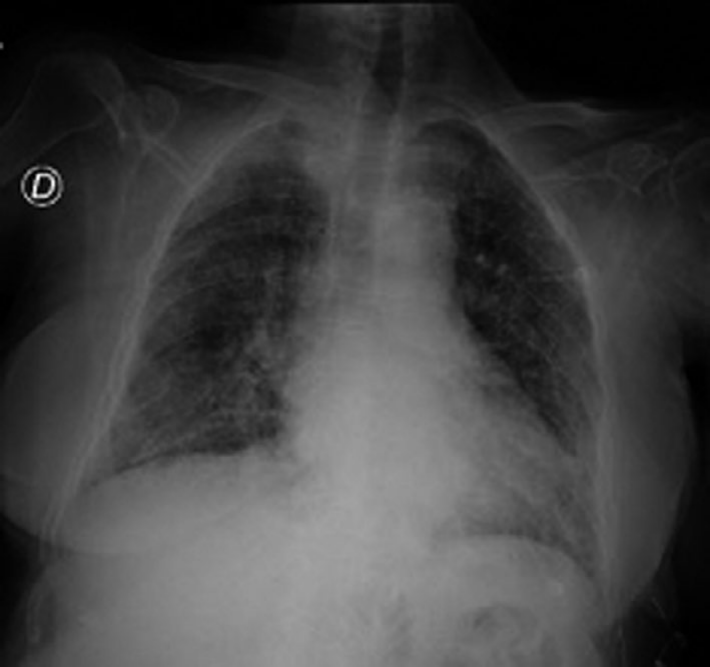
Chest X‐rays performed on the day of admission

On admission, physical examination showed decreased breath sound, diffuse systolic murmur, and time‐space disorientation. Respiratory rate was 26 acts per minute. Her hemato‐biochemical examinations were unremarkable. Arterial oxygen saturation (SpO2) was 85% at rest in ambient air. She began therapy with low‐flow oxygen support at 7 L/min. An hour later, arterial blood gas analysis (ABG) showed a P/F ratio of 250 indicating mild respiratory failure. Since the patient was not compliant with nasal cannula, which was self‐removed several times, SpO2 deteriorated, so a Venturi Mask with FiO2 60% was positioned. Follow‐up ABG showed P/F ratio of 153. Chest X‐rays performed on the same day of admission showed diffuse interstitial thickening (Figure 1).

The patient was prescribed the following therapy: methylprednisolone, hydroxychloroquine, azithromycin, enoxaparin, and n‐acetyl cysteine.

**FIGURE 2:**
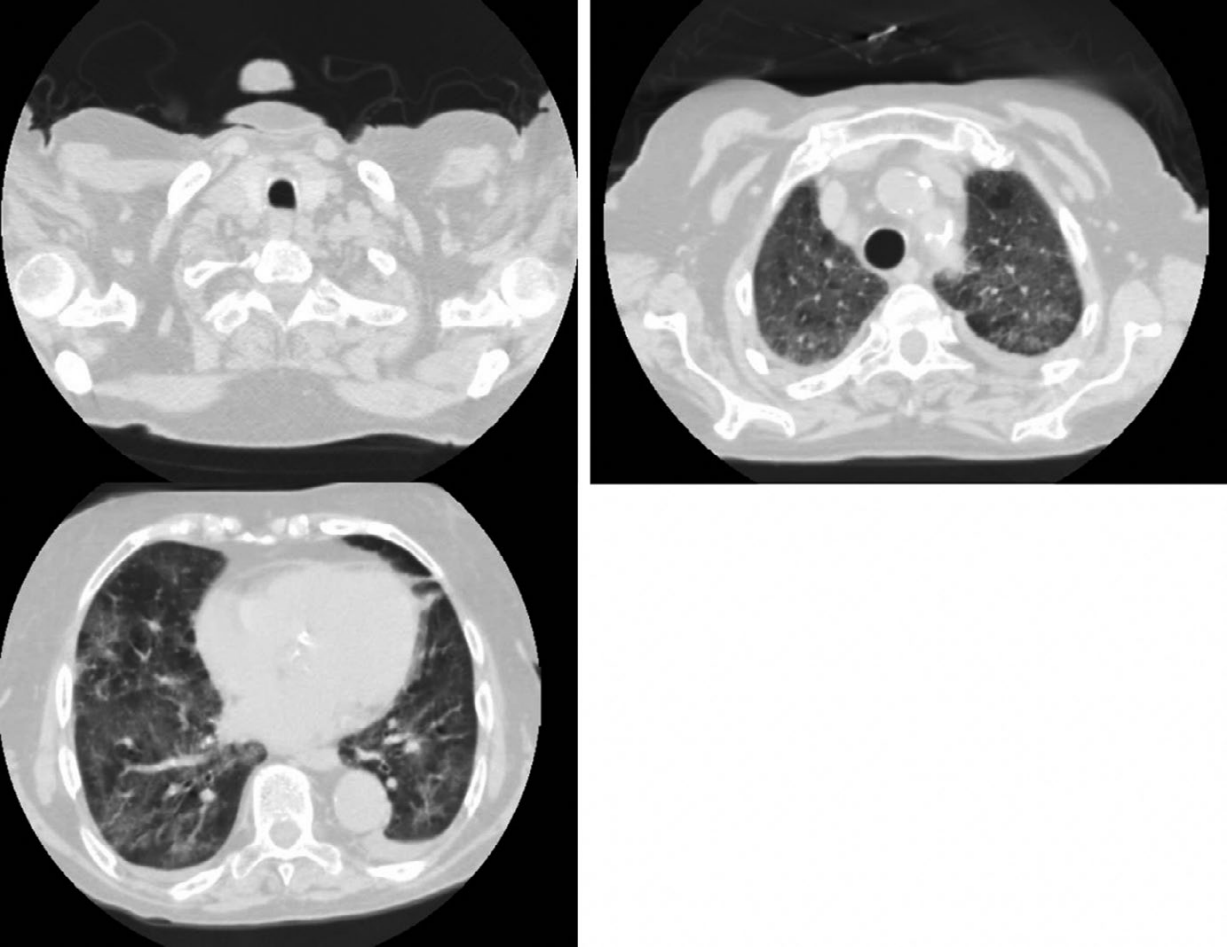
High‐resolution computerized tomography performed on 4th day of hospitalization

On the 4th day of hospitalization, the patient conditions worsened notwithstanding the respiratory support and treatment applied, with a P/F ratio of 85.5. Therefore, we decided to start oxygen therapy with HFNC (FiO2: 72%, flow: 40 L/min with SpO2: 96%). The same day, high‐resolution CT scan documented widespread ground‐glass opacities with diffuse interstitial thickening and crazy paving (Figure 2).

**FIGURE 3:**
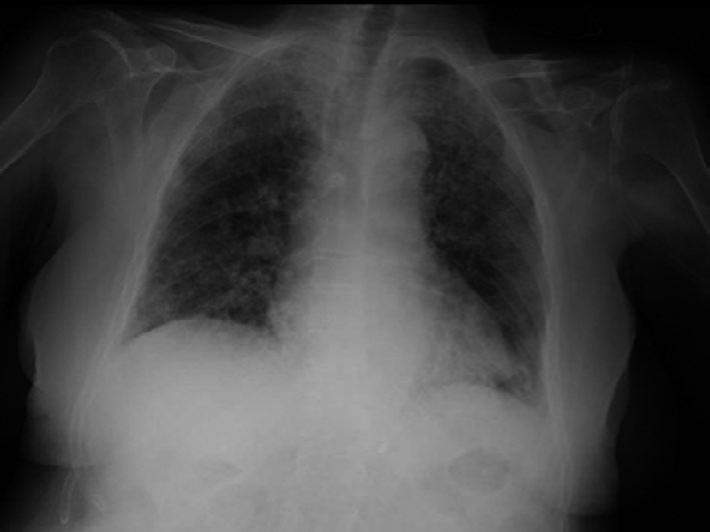
Chest X‐rays performed on the 14th day of hospitalization

Respiratory function improved slightly under prolonged support with HFNC. In fact, P/F ratio was 126. Chest X‐rays performed on the 14th day of hospitalization showed a small improvement if compared to the previous (Figure 3).

During the following days, the patient's conditions improved further, so FiO_2_ was titrated to 36%, maintaining SpO_2_ at 97%, keeping a flow of 45 L/min.

**FIGURE 4:**
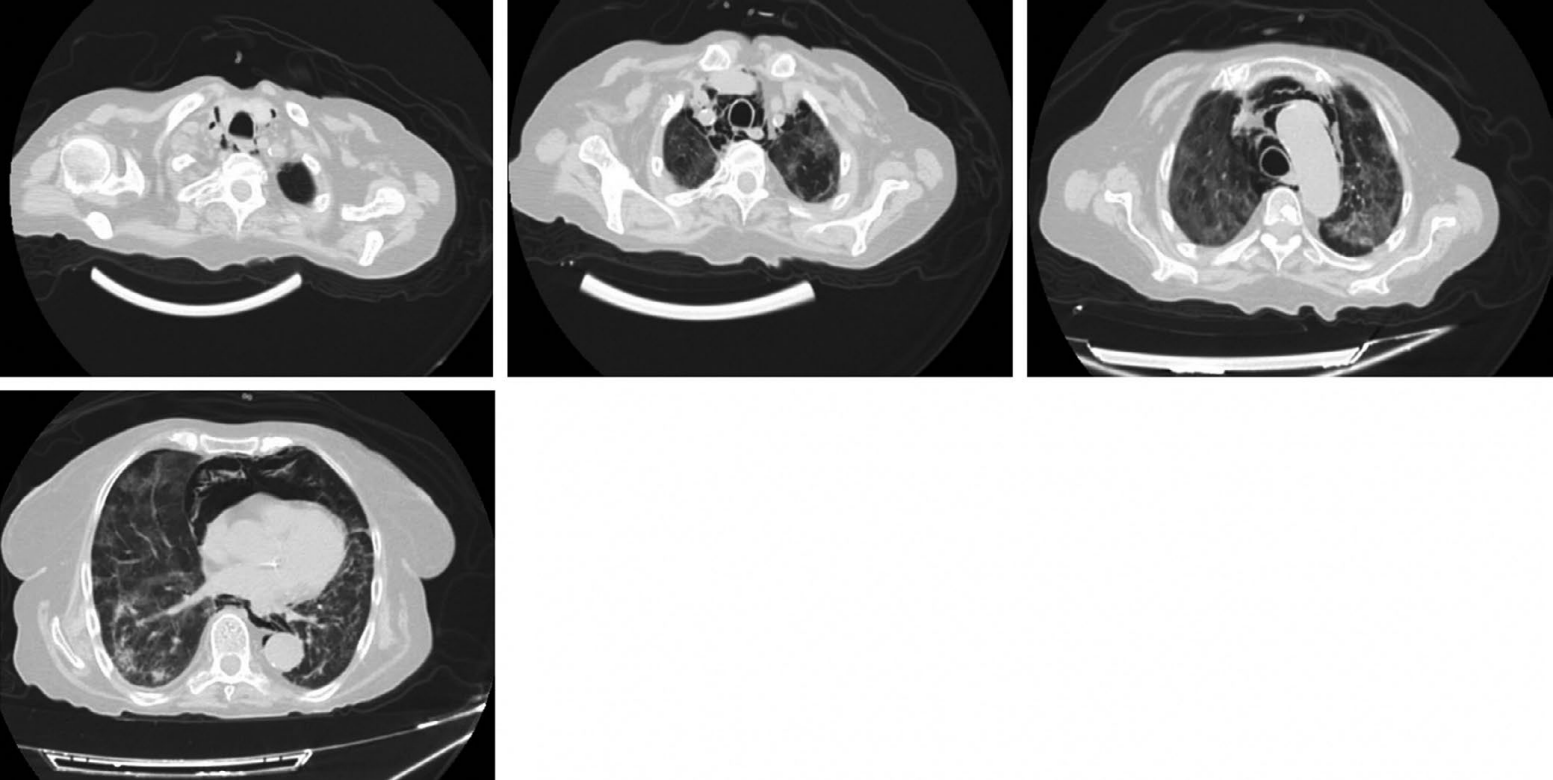
Computerized tomography performed on the 21st day of hospitalization

**FIGURE 5:**
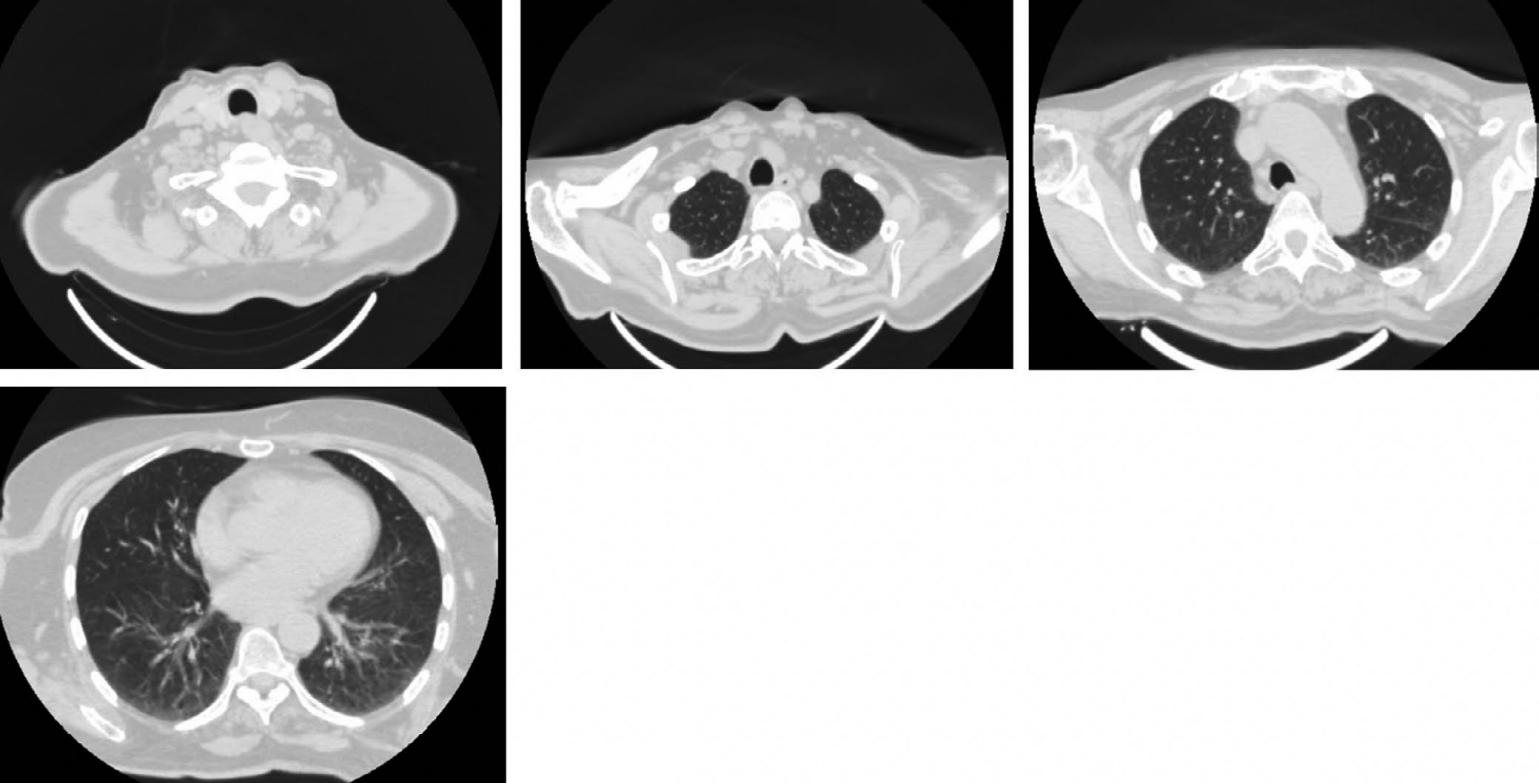
High‐resolution computerized tomography performed after 38 days of hospitalization, 17 days after the onset of pneumomediastinum

Despite this improvement, on the 21st day of hospitalization, the patient complained of dyspnea and chest pain, which increased with respiratory acts and radiated to the neck, especially along the left side. An electrocardiogram was performed, not supporting myocardial infarction, as well as cardiac enzymes which were normal. Physical examination showed decreased breath sounds; the percussion was hyper‐resonant with no evidence of coarse crackles excluding a pulmonary edema. PCR, procalcitonin, and WBC were negative, and there were no signs of bacterial pneumonia. Arterial oxygen saturation improved, so FiO_2_ was reduced down to 21%, maintaining a flow of 45 L/min. Due to dyspnea and D‐dimer increase up to 1.02 mg/L (normal range: 0 to 0.55 mg/L) in the suspicion of thromboembolism as a possible differential diagnosis in our patient, we performed a CT scan, including the study of the pulmonary arteries. This examination showed extensive pneumomediastinum, subcutaneous emphysema, and pneumothorax without evidence of any embolisms (Figure 4). In the presence of pneumomediastinum and improvement of gas exchanges, we decided to use a low‐flow system such as nasal cannulas. The patient maintained hemodynamic stability. After 1 month from the admission, the patient was eupneic without any signs of respiratory failure with good gas exchanges without oxygen support and chest pain also disappeared. High‐resolution CT scan performed after 38 days of hospitalization documented complete resolution of the pneumomediastinum (Figure 5). The patient was therefore discharged in good conditions.

## 3 CASE REVIEW OF THE LITERATURE

A systematic review of the literature for all relevant articles was performed using PubMed. Articles were limited to those published in the English language. The search strategy used the keywords and MeSH terms: “pneumomediastinum”, “mediastinal emphysema”, “COVID‐19”, “SARS‐CoV‐2”.

Up to the date of submission of the present manuscript, a total of 41 cases of pneumomediastinum have been reported in patients suffering from COVID‐19,^8^, ^23^ whose characteristics are summarized in Table 1. Among these 41 patients, 18 (44%) had been previously treated with invasive mechanical ventilation and one with noninvasive ventilation (NIV), thus suggesting a possible iatrogenic nature of pneumomediastinum. Only in one case, pneumomediastinum occurred in a patient after HFNC but subsequently increased in extent with pneumothorax occurring after NIV.^11^ In the remaining cases reported so far, pneumomediastinum appeared to occur independently from mechanical ventilation (Table 1).

**TABLE 1:** Summary of cases of pneumomediastinum in patients with COVID‐19

Age (years)	Gender	Respiratory support prior to pneumomediastinum	Treatment	Outcome^a^	Reference
23	F	None	Azithromycin Chloroquine	Positive	Kolani et al^6^
38	M	HFNC prior to pneumomediastinum and then NIV	/	Not reported	Sun et al^9^
38	M	Oxygen	Moxifloxacin Ribavirin methylprednisolone Theophylline ambroxol Cefoperazone‐tazobactam Recombinant human interferon alpha‐1b via aerosol	Positive	Zhou et al^10^
64	M	Oxygen via nasal cannula	Not reported	Positive	Goldman et al^11^
30	M	Not reported	Not reported	Positive	Romano et al^12^
65	M	Not reported	Not reported	Positive	
84	F	Oxygen mask with reservoir	Hydroxychloroquine Ceftriaxone Methylprednisolone	Negative	Vega et al^13^
67	M	Oxygen mask	Piperacillin/tazobactam Azithromycin	Negative	
73	M	Oxygen mask with reservoir prior to pneumomediastinum, and then CPAP	Hydroxychloroquine Azithromycin Tocilizumab Methylprednisolone	Negative	
49	M	Non‐rebreather oxygen mask	Ceftriaxone doxycycline steroids Enoxaparin sodium hydroxychloroquine	Positive	Mohan et al^14^
65	M	Oxygen via nasal cannula or oxygen mask with reservoir	Azithromycin Hydroxychloroquine	Positive	Gorospe et al^15^
60	M		Hydroxychloroquine, Lopinavir/ritonavir Tocilizumab Steroids	Negative	
62	F		Hydroxychloroquine Azithromycin Lopinavir/Ritonavir Tocilizumab	Positive	
58	F		Azithromycin Hydroxychloroquine Steroids	Positive	
55	F	Oxygen mask with reservoir	Azithromycin Ceftriaxone Hydrocortisone	Negative	Quincho‐Lopez et al^16^
31	M	Oxygen nasal cannula	Azithromycin Ceftriaxone Hydrocortisone	Positive	
Not reported	Not reported	10 patients: Mechanical ventilation	Not reported	Negative	Eperjesiova et al^17^
Not reported	Not reported	5 patients without Oxygen support, of which 3 required eventually mechanical ventilation	Not reported	4 positive 1 negative	
36	M	After the onset of pneumomediastinum: Mechanical ventilation Pronation Veno‐venous extracorporeal membrane oxygenation	Azithromycin Vitamin C Zinc sulfate Hydroxychloroquine Tocilizumab Solumedrol	Negative	Al Azzawi et al^18^
47	M	Non‐rebreather oxygen mask High‐flow nasal cannula Mechanical ventilation Pronation	Hydroxychloroquine Azithromycin Zinc sulfate Tocilizumab	Positive	
78	M	Mechanical ventilation	Hydroxychloroquine Azithromycin Zinc sulfate Norepinephrine	Negative	
67	M	High‐flow nasal cannula Mechanical ventilation Pronation	Antibiotics Antivirals Vasoconstrictors	Negative	Xiang et al^19^
Median (range) age was 60 years (38‐70 years)	M	5 patients: Mechanical ventilation	Not reported	2 Negatives 3 Positives	Wali et al^20^
36	F	Oxygen mask NIV	Antivirals Anti‐inflammatory drugs	Negative	Wang et al^21^

^a^ The outcome is considered to be positive when the patient survived and negative when the patient died.

## 4 DISCUSSION

We present herein a case of pneumomediastinum in a patient with acute respiratory failure related to COVID‐19. The patient was treated with HFNC, which appeared to be beneficial, but nonetheless, it could have triggered pneumomediastinum. To our best knowledge, this is the second case of pneumomediastinum occurring after HFNC in a COVID‐19 patient (see Table 1). However, it is difficult to attribute pneumomediastinum to a single cause in our patient.

Spontaneous pneumomediastinum is defined as air in the mediastinum without evident causes (eg, traumatic, iatrogenic, hollow organ perforation, gas‐producing infection surgery, or any other interventions).^8^ Numerous reports^24^, ^25^, ^26^, ^27^, ^28^, ^29^, ^30^, ^31^, ^32^, ^33^, ^34^, ^35^, ^36^, ^37^, ^38^, ^39^, ^40^, ^41^, ^42^, ^43^, ^44^, ^45^, ^46^, ^47^, ^48^, ^49^, ^50^, ^51^, ^52^, ^53^, ^54^, ^55^, ^56^ have demonstrated that several lung infections, mainly influenza, provoke pneumomediastinum. Radiological features are different in influenza pneumonia compared with COVID‐19 and pneumomediastinum is believed to be a much less frequent complication (or never occurs) in COVID‐19 pneumonia.^31^, ^32^, ^35^, ^37^, ^57^ By contrast, our case report indicates that it may occur, albeit this complication may have been favored by HFNC and other conditions.

Although pneumomediastinum was reported as a complication of several lung infections,^24^, ^25^, ^26^, ^27^, ^28^, ^29^, ^30^, ^31^, ^32^, ^33^, ^34^, ^35^, ^36^, ^37^, ^38^, ^39^, ^40^, ^41^, ^42^, ^43^, ^44^, ^45^, ^46^, ^47^, ^48^, ^49^, ^50^, ^51^, ^52^, ^53^, ^54^, ^55^, ^56^ it is possible that alterations induced by SARS‐CoV‐2 infection are more complex. For instance, SARS‐CoV‐2 infects both type I and II pneumocytes, and this may cause damage of the integrity of the alveolar membrane, which may lead to alveolar rupture.^8^ Moreover, in addition to a direct damage provoked by SARS‐CoV‐2 infection, indirect mechanisms due to cytokine storm^3^ or micro‐ and macrothrombosis events^58^ may play a role. It is currently unknown whether the increase in the transalveolar pressure during COVID‐19 is greater than that occurring during other infections.

Mechanisms of action of HFNC are original and complex. In fact, HFNC delivers a mixture of air and oxygen, heated and humidified, at a higher flow than the patient's inspiratory peak. Thus, a further withdrawal of air from outside is not necessary to adjust the flow administered and required by the patient, contrary to what is obtained with low‐flow systems.^59^ Consequently, the only inhaled oxygen mixture is that coming from the nasal cannulas. Therefore, the oxygen concentration (FiO2) set on the device corresponds to that inhaled by the subject. Furthermore, HFNC displays a beneficial effect on CO2 washout at high flow rates and on the alveolar recruitment.^60^ As a form of oxygen support, HFNC is considered to be safe and gentler than mechanical ventilation when used in adults with moderate hypoxemia,^61^, ^62^ and Vianello et al^63^ tested HFNC in patients with severe hypoxemic acute respiratory failure consequent to SARS‐CoV‐2 infection, suggesting its safety for less severe patients. However, they were rarely reported, pneumothorax and pneumomediastinum are possible complications of HFNC even in non–COVID‐19 patients.^64^, ^65^, ^66^

An increased gradient of pressure in the alveoli compared with the lung interstitium was described as a possible mechanism by Makclin et al.^67^ Following rupture of the alveolar membrane, air penetrates into the interstitium, first causing interstitial emphysema, subsequently reaching the pulmonary hilum, and eventually provoking pneumomediastinum.^68^ This can happen when the intra‐alveolar pressure increases or when the pressure in the interstitium decreases. The former mechanism occurs in those conditions mimicking the Valsalva maneuver, such as coughing or sneezing, while the latter mechanism occurs during extreme respiratory effort, marijuana smoking, diabetic ketosis, or rapid reduction in atmospheric pressure.^69^ In our patient, not only HFNC but also coughing could explain the increase in intra‐alveolar pressure. In other terms, we cannot exclude that pneumomediastinum was from cough‐induced barotrauma rather than from HFNC in our patient. It is, however, possible that, in patients with a more severe alveolar damage due to SARS‐CoV‐2 infection, and with precipitating factors such as cough, HFNC increases the risk of pneumomediastinum.

Lastly, we performed a review of patients with pneumomediastinum and found that approximately half of the cases were mechanically ventilated and half were not (see Table 1), possibly indicating that mechanical ventilation is a main risk factor, while HFNC may be better tolerated. Mediastinal emphysema and pneumothorax are well‐known complications of mechanical ventilation. In fact, positive pressure mechanical ventilation may lead to pulmonary barotrauma due to elevation of the transalveolar pressure which is the difference in pressure between the alveolar pressure and the pressure in the interstitial space.^70^

Volutrauma is referred to an excessive strain and may occur when tidal volume is set above 12 mL/kg predicted body weight, but decreases in tidal volume below 6 mL/kg, as proposed for “ultraprotective ventilation,” would not necessarily be of benefit.^71^ It has to be seen whether, with increasing amount of patients under observation and increasing utilization of HFNC, pneumomediastinum will appear with more frequency. Also, further studies should determine what impact HFNC will have compared with different techniques of respiratory support and with different patient conditions.

## 5 CONCLUSIONS

We described herein a case of pneumomediastinum in a COVID‐19 patient who was treated with HFNC. Several mechanisms could explain the occurrence of pneumomediastinum in our patient, as well as in other cases reported so far. So, we question whether it is appropriate to define such cases of pneumomediastinum as truly “spontaneous.” Oxygen support with HFNC is gentler than mechanical ventilation and may provide significant benefits, but more studies are needed to investigate the efficacy and safety of different respiratory supports in patients with COVID‐19 pneumonia in order to provide more evidence‐based recommendations. Moreover, it is important to recognize that an alternate to HFNC in COVID‐19 patients, to prevent pneumomediastinum, may not be feasible because both HFNC and ventilation strategies increase the risk of barotrauma and alveolar rupture.

Meanwhile, a prompt diagnosis of pneumomediastinum should be made if chest pain and/or dyspnea occur in these patients, since this may have a great impact on patient prognosis.^72^, ^73^, ^74^

## CONFLICT OF INTEREST

All authors declare that they have no conflict of interest in the publication of this paper. This study did not receive any specific grants from any funding agencies in the public, commercial, and nonprofit sectors. Dr Maria Mazzitelli was supported as PhD student by European Commission (FESR FSE 2014‐2020) and by Calabria Region (Italy). European Commission and Calabria Region cannot be held responsible for any use, which may be made of information contained therein.

## AUTHOR CONTRIBUTIONS

Anna Cancelliere and Giada Procopio: wrote the original draft, conceptualized the study, designed the methodology, and performed data curation. Elena Lio: designed the methodology and performed data curation. Maria Mazzitelli, Maria Petullà, Francesca Serapide, Maria Chiara, Pelle, and Chiara Davoli: performed data curation. Enrico Maria Trecarichi, Carlo Torti: reviewed the manuscript and supervised the study.

## ETHICAL APPROVAL

Local Ethical Committee approved retrospective collection of data and patient signed informed consent for publication.

## Data Availability

All available data are reported in the present manuscript.
